# Surgical site infections after cesarean delivery: epidemiology, prevention and treatment

**DOI:** 10.1186/s40748-017-0051-3

**Published:** 2017-07-05

**Authors:** Tetsuya Kawakita, Helain J. Landy

**Affiliations:** 1 Obstetrics and Gynecology, MedStar Washington Hospital Center, 101 Irving Street, 5B45, NW, Washington, DC 20010 USA; 2 Obstetrics and Gynecology, MedStar Georgetown University Hospital, Washington, DC USA

**Keywords:** Cesarean delivery, Chlorhexidine skin preparation, Surgical bundle, Surgical site infection, Vaginal cleansing

## Abstract

Cesarean delivery (CD) is one of the most common procedures performed in the United States, accounting for 32% of all deliveries. Postpartum surgical site infection (SSI), wound infection and endometritis is a major cause of prolonged hospital stay and poses a burden to the health care system. SSIs complicate a significant number of patients who undergo CD – 2-7% will experience sound infections and 2-16% will develop endometritis. Many risk factors for SSI have been described. These include maternal factors (such as tobacco use; limited prenatal care; obesity; corticosteroid use; nulliparity; twin gestations; and previous CD), intrapartum and operative factors (such as chorioamnionitis; premature rupture of membranes; prolonged rupture of membranes; prolonged labor, particularly prolonged second stage; large incision length; subcutaneous tissue thickness > 3 cm; subcutaneous hematoma; lack of antibiotic prophylaxis; emergency delivery; and excessive blood loss), and obstetrical care on the teaching service of an academic institution. Effective interventions to decrease surgical site infection include prophylactic antibiotic use (preoperative first generation cephalosporin and intravenous azithromycin), chlorhexidine skin preparation instead of iodine, hair removal using clippers instead of razors, vaginal cleansing by povidone-iodine, placental removal by traction of the umbilical cord instead of by manual removal, suture closure of subcutaneous tissue if the wound thickness is >2 cm, and skin closure with sutures instead of with staples. Implementation of surgical bundles in non-obstetric patients has been promising., Creating a similar patient care bundle comprised evidence-based elements in patients who undergo CD may decrease the incidence of this major complication. Each hospital has the opportunity to create its own CD surgical bundle to decrease surgical site infection.

## ﻿Background﻿

Cesarean delivery (CD) is one of the most common procedures performed in the United States, accounting for 32% of all deliveries [1]. In 2014, nearly 1.3 million CDs were performed [1]. As with all surgical procedures, CD can be associated with SSIs, including wound infections and endometritis, as well as being associated with higher maternal morbidity and mortality with future pregnancies [2–5].

## Epidemiology

### Wound complications

#### Wound hematoma, seroma, dehiscence

Wound hematoma and seroma are collections of blood and serum, respectively. Hematomas are usually due to failure of primary hemostasis or bleeding diathesis such as anticoagulation therapy. Vigorous coughing or severe hypertension immediately after surgery may contribute to the formation of hematoma. Wound hematoma or seroma, described in 2-5% of women after CD can cause wound dehiscence and act as a nidus for development of wound infection [6, 7]. Wound dehiscence is separation of incision and complicates 2-7% after CD [6, 7].

#### Wound infection

Wound infection presents with erythema, discharge, and induration of the incision, complicates 2-7% of patients and generally develops 4 to 7 days after CD [8–12]. When wound infection develops within 48 h, the offending organisms usually are groups A or B-hemolytic Streptococcus. Other common pathogens involved in wound infections are Ureaplasma urealyticum, *Staphylococcus epidermidis*, Enterococcus facialis, *Staphylococcus aureus*, *Escherichia coli*, and Proteus mirabilis [13, 14].

#### Necrotizing fasciitis

Necrotizing fasciitis is a rare but serious infection causing significant morbidity after CD that is characterized by rapid and progressive necrosis of subcutaneous tissue and fascia [15]. Necrotizing fasciitis is suspected with severe pain, crepitus, wooden-hard induration of the subcutaneous tissues, bullous lesions, skin necrosis or ecchymosis, and elevated serum creatine kinase level; a hallmark of necrotizing fasciitis is rapid progression of clinical manifestations [16–18]. Imaging studies such as computed tomography or magnetic resonance imaging may show edema extending along the fascial plane. The diagnosis is confirmed at the time of repeat surgery. Features suggestive include fascia that swollen and dull gray in appearance with areas of necrosis, skin necrosis with easy dissection along the fascia, or presence of gas in the soft tissues.

Type I necrotizing fasciitis results from a polymicrobial infection involving both aerobic and anaerobic bacteria; type II necrotizing fasciitis is generally caused by a single organism, group A streptococcus. In a 1997 study, necrotizing fasciitis occurred in only 0.18% of women who underwent CD; the onset occurred between 5 and 17 days following CD. The authors reported a very high mortality rate (22%) [15], which implied the importance of prompt recognition and treatment of necrotizing fasciitis.

### Endometritis

Postpartum endometritis results from a polymicrobial infection of the decidua, characterized by fever ≥38.0 °C, fundal tenderness, and purulent discharge from the uterus [19]. Higher risks for endometritis are associated with CD compared to vaginal delivery [5]. Postpartum endometritis complicates 2-16% of women who underwent CD [8–12]. Risks are higher following CD performed in labor (3-11%) compared with prelabor CDs (0.5-5%), as well as in patients who had ruptured membranes compared to intact membranes (3-15% vs. 1-5%, respectively) [10, 20, 21]

### Risk factors for surgical site infections following CD

Many different fisk factors for SSIs following CD have been reported (Table 1). In decreasing order of significant risk as measured by relative risks or odds ratios, risk factors include subcutaneous hematoma [8], chorioamnionitis [22–24], maternal comorbidities (American Society of Anesthesiologists class of 3 or greater) [22], tobacco use in pregnancy [23], incision length > 16.6 cm [25], limited prenatal care (fewer than 7 visits) [26], body mass index >30 or 35 kg/m^2^ [22, 24, 25, 27–29] corticosteroid use [25], subcutaneous tissue thickness > 3 cm [30], prolonged second stage (compared with first stage) [31], teaching service [8], no antibiotic prophylaxis [26], pregestational diabetes [27, 29, 32], operating time ≥ 38 min [28], hypertensive disease/preeclampsia [22, 29], duration of labor >12 h [24], nulliparity [22], twin gestations [29], premature rupture of membranes [29], gestational diabetes [33], blood loss (increased for every increase in blood loss of 100 mL) [22], previous cesarean delivery [33], emergency delivery [29], and rupture of membranes (increased risk for every additional hour) [26].

**Table 1:** Risk factors for surgical site infection

Variables	Relative risk or odds ratios	References
Subcutaneous hematoma	11.6	8
Chorioamnionitis	5.6-10.6	21-23
American Society of Anesthesiologists class of 3 or greater	5.3	21
Tabacco	5.3	22
Incision length > 16.6 cm	4.9	24
Prenatal visit <7	4	25
Body Mass Index >35 kg/m2	3.7	26
Corticosteroid	3.1	24
Body Mass Index >30 kg/m2	2.0-2.8	21, 23, 24, 26-28
Subcutaneous tissue thickness > 3 cm	2.8	29
Second stage (vs. first stage)	2.8	30
Teaching service	2.7	8
No antibiotic prophylaxis	2.6	25
Pregestational Diabetes	1.4-2.5	26, 28, 31
Operating time ≥ 38 min	2.4	27
Hypertensive disease/Preeclampsia	1.7-2.3	21, 28
Duration of labor >12 h	2.0	23
Nulliparity	1.8	21
Twin	1.6	28
Premature rupture of membrane	1.5	28
Gestational diabetes	1.5	32
Blood loss (every 100 ml)	1.3	21
Previous cesarean delivery	1.3	32
Emergency delivery	1.3	28
Rupture of mambranes (each hour)	1.02	25

### Burden to healthcare system

Postpartum infections are a major cause of prolonged hospital stay and comprise a large burden to our health care system [12]. One study attributed costs of an additional $3700 for wound infection and an additional $4000 for endometritis (in 2008 US dollars, corresponding to $4200 and $4500 today, respectively) [34].

## Prevention

Previous studies have shown that certain interventions lower SSIs. Since CD is one of the most common procedures performed worldwide, it is important for implementation of evidence-based approaches to decrease such postoperative complications. A summary of studies describing such approaches is presented in Table 2.

**Table 2:** Interventions and techniques surrounding cesarean delivery

	Wound infection		Endometritis	
Variables	RR or OR	References	RR or OR	References
Preoperative elements				
First generation cephalosporin vs. none	0.38	34	0.42	34
First generation cephalosporin prior to skin incision vs. after cord clamp	0.7	11, 35-38	0.21-0.61	11, 35-38
First generation cephalosporin 2 g vs. 3 g in morbidly obese women	NS	40	-	-
Azithromycin				
In labor	0.35	9	0.62	9
Non labor	-	-	0.11	41
Chlorhexidine alcohol skin preparation vs. iodine	0.55	42, 43	NS	42
Razor hair removal vs. clippers	2.1	44	-	-
Vaginal cleansing				
Chlorhexidine vs none	NS	45	0.2	45
Iodine vs none	NS	10, 18, 46, 47	0.39	10, 18, 46, 47
In labor	NS	47	NS	47
Not in labor	NS	47	NS	47
Ruptured membranes	NS	46, 47	0.13	46, 47
Intact membranes	NS	47	NS	47
Intraoperative elements				
Uterine exteriorization	NS	49	NS	49
Manual removal of placneta vs. traction of umbilical cord	-	-	1.4-1.6	50, 51
Intraabdominal irrigation	NS	53	NS	52, 53
Closure of subcutaneous tissue if >2 cm^a^	NS	54	-	-
Subcutaneous drain	NS	55, 56	-	-
Suture skin closure vs. staple^b^	NS	6, 57, 58	-	-
Postoperative elements				
Dressing removal between 24 and 48 h vs. 6 h	NS	7	-	-

*RR* relative risk; *OR* odds ratio; *NS* not statistically significant; *CI* confidence interval

^a^Wound complications (hematoma, seroma, and infection) - RR 0.66; 95%CI 0.48, 0.91; Wound separation - RR 0.42; 95%CI 0.24, 0.75

^b^Wound complications (wound infection, hematoma, seroma, or separation of 1 cm or longer) -adjusted OR 0.43; 95%CI 0.23, 0.78; wound separation - adjusted OR 0.20; 95%CI 0.07, 0.51

## Preoperative management

### Preoperative antibiotics

#### Cefazolin

Administration of a first generation cephalosporin is the mainstay of the prevention of SSIs after CD. A meta-analysis of randomized controlled trials showed that the use of first generation cephalosporin compared with no antibiotics decreased the risks for development of wound infections (Relative Risk [RR] 0.38; 95% confidence interval [CI] 0.28, 0.53) and endometritis (RR 0.42; 95% CI 0.33, 0.54) [35]. Further, lower rates of SSIs have been found with antibiotic administration of first generation cephalosporin prior to skin incision compared with administration after cord clamp [11, 36–39]. The meta-analysis by Constantine et al. reported the risk of endometritis was lowered significantly (RR 0.47; 95% CI 0.26, 0.85) [11]. Another publication of a randomized controlled trial showed lower rates of both wound infection (OR] 0.7; 95% CI 0.55, 0.90) and endometritis (OR 0.61; 95% CI 0.47, 0.79) when antibiotic prophylaxis was given prior to skin incision compared with after cord clamp [39].

The American College of Obstetricians and Gynecologists (ACOG) recommend infusion of intravenous 1 g cefazolin within 60 min prior to skin incision [40]. For women with (BMI >30 kg/m^2^ or weight > 100 kg, a dose of 2 g cefazolin intravenous infusion is recommended [40]. Though use of higher doses have been considered in women with BMI > 40 kg/m^2^, one retrospective study of morbidly obese women did not demonstrate a difference in SSIs comparing cefazolin doses of 2 g and 3 g [41].

#### Azithromycin

Recent reports have shown benefits to adding azithromycin at the time of CD. A 2008 study by Tita et al. described a lower risk of endometritis with the routine use of intravenous azithromycin compared to standard antibiotic prophylaxis (0.9% vs. 12.5%; RR 0.11; 95% CI 0.06, 0.19; *P* < .001) [42]. In 2016, this same author reported that adding intravenous azithromycin 500 mg to standard preoperative antibiotic prophylaxis was associated with lower risks of endometritis (3.8% vs. 6.1%; RR 0.62; 95% CI 0.42, 0.92; *P* = .02) and wound infection (2.4% vs. 6.6%; RR 0.35; 95% CI 0.22, 0.56; *P* < .001) in women undergoing non-elective CD compared with placebo. [9] The addition of preoperative azithromycin does not have any short term effects on the neonates though long term data are lacking. The randomized controlled study of azithromycin in addition to standard antibiotics did not demonstrate any differences in composite neonatal outcome, death, and NICU admission [9].

### Chlorhexidine alcohol skin preparation

Skin preparation (chlorhexidine alcohol vs. iodine) has been examined by 2 randomized controlled trials with disparate results. [43, 44] Tuuli et al. compared 572 women with chlorhexidine skin preparation with 575 women with iodine skin preparation in a randomized controlled study and demonstrated a decreased rate of wound infection (RR 0.55; 95%CI 0.34, 0.90; *P* = .02) in patients with chlorhexidine skin preparation [43]. Results from another randomized controlled trial comparing povidone iodine alone (*n* = 463), chlorhexidine alone (*n* = 474), and both (*n* = 467) showed similar wound infection rates, however [44]. The reason for of the different results of these two randomized controlled trials is unclear. Nonetheless, a shift has begun towards chlorhexidine alcohol and away from povidone iodine skin preparation [18].

### Use of clippers instead of razor

A 2011 Cochrane Data Base meta-analysis by Tanner et al. comparing hair removal by shaving with clipping showed a higher risk of wound infection associated with shaving (RR 2.09; 95%CI 1.15, 3.80) [45]. Use of clippers instead of razor for preoperative hair removal has been adopted by many hospitals.

### Preoperative vaginal cleansing

There has been a growing interest in assessing potential benefits from preoperative vaginal cleansing. In the randomized controlled trial of 218 women comparing chlorhexidine vaginal cleansing with no vaginal cleansing by Ahmed et al., chlorhexidine vaginal cleansing compared with no vaginal cleansing was associated with a lower rate of endometritis (RR 0.2; 95% CI 0.06, 0.7) although the rate of wound infection was similar (RR 0.6; 95% CI 0.2, 1.8) [46]. Vaginal cleansing by povidone iodine also has been studied [10, 20, 47, 48]. Similar results were reported using povidone-iodine in the Cochrane Data Base meta-analysis by Haas in which vaginal preparation by povidone-iodine compared with no preparation demonstrated a lower risk of endometritis (RR 0.39; 95% CI 0.16, 0.97) with similar risks of wound infection (RR 0.99; 95% CI 0.57, 1.70) [48]. The risk reduction was particularly strong in women with ruptured membranes who had vaginal preparation by povidone-iodine compared with no preparation (RR 0.13; 95% CI 0.02, 0.66) [48].

Currently, only povidone-iodine is approved for use in the vagina, though off-label use of chlorhexidine solutions can be considered, especially in women with allergies to iodine [49]. The use of preoperative vaginal cleansing should be considered, especially in women with ruptured membranes.

## Intraoperative management

Many surgical maneuvers have been taught over time without the benefit of evidence-based medicine. Below are a number of such intraoperative steps that have been evaluated recently for their merit.

### Uterine exteriorization

Exteriorization of the uterus at the time of CD is often performed for better visualization to repair the uterine incision. A meta-analysis comparing repair in situ and by uterine exteriorization did not demonstrate any statistically significant differences in surgical time, intraoperative nausea or vomiting, endometritis, or wound infection [50]. The decision on whether or not to exteriorize the uterus should depend on provider preference.

### Removal of placenta by traction of umbilical cord

Manual removal of the placenta compared with removal via umbilical cord traction has been associated with endometritis [51, 52]. In one meta-analysis, manual removal of the placenta was associated with a higher risk of endometritis compared with traction of umbilical cord (RR 1.64, 95% CI 1.42, 1.90) [51].

### Intraabdominal irrigation

Two studies have not demonstrated a reduction of SSIs with intraabdominal irrigation of normal saline [53, 54]. In a randomized controlled trial of 236 women undergoing CD, intraabdominal irrigation did not demonstrate decreased risks of wound infection and endometritis, but was associated with intraoperative nausea (RR 1.62; 95% CI 1.15, 2.28) [53]. Similarly in a randomized controlled trial of 196 women undergoing CD, intraabdominal irrigation by normal saline did not reduce intrapartum or postpartum maternal morbidity [54]. Evidence does not support use of routine intraabdominal irrigation.

### Suture closure of subcutaneous tissue if wound thickness greater than 2 cm

In a meta-analysis of randomized controlled trials, Chelmow et al. evaluated the potential benefit of suture closure of subcutaneous tissue relative to tissue thickness [55]. Their study showed a statistically significant decrease in the rate of wound complications when the subcutaneous thickness was greater than 2 cm (RR 0.66; 95% CI 0.48, 0.91). [55]. This group also found that suture closure of subcutaneous tissue was associated with a lower risk of seroma (RR 0.42; 95% CI 0.24,1.49) but not lower risks of wound hematomas (RR 1.03; 95% CI 0.38, 2.76) or wound infections (RR 0.98; 95% CI 0.65, 1.49) [55]. Current practice has adopted use of subcutaneous closure when the subcutaneous thickness measures greater than 2 cm.

### Subcutaneous drain

Ramsey et al. reported a randomized controlled trial of 280 women with subcutaneous thickness 4 cm or more showing that routine subcutaneous drain was not associated with wound complications compared with standard suture reaproximation [56]. This finding was confirmed by a meta-analysis [57]. Routine subcutaneous drain is not recommended.

### Suture skin closure instead of staple closure

Studies comparing suture and staple closure of the skin after CD are consistent in advising use of sutures over staples. In 2011, Tuuli et al. reported a meta-analysis of 6 studies and showed that an increased risk of wound infection or wound separation with staples (*n* = 803) compared with suture skin closure (*n* = 684) (OR 2.06; 95% CI 1.43, 2.98) [58]. A 2012 Cochrane Review of 18 trials by Mackeen et al. showed no increased risk of wound infection with staple skin closure [59]. Subsequently in 2014, a randomized controlled study of 746 women by the same group showed a lower risk of wound complications (wound infection, hematoma, seroma, or separation of 1 cm or longer) with suture skin closure compared with staple skin closure (adjusted OR 0.43; 95% CI 0.23, 0.78) [6]. In this last study, the lower rate of wound complications was largely due to a decreased incidence of wound separation (adjusted OR 0.20; 95% CI 0.07, 0.51). Based on this evidence, routine staple skin closure is not recommended. Further studies are needed to answer if decreased risk of wound complications in suture skin closure compared with staple closure is reproducible in vertical skin incision.

### Prophylactic negative pressure wound therapy

Negative pressure wound therapy (vacuum-assisted wound closure) after CD is gaining popularity, especially in obese women. Negative pressure reduces excess fluid accumulation and protects the wound from irritation caused by reducing the frequency of dressing changes to only every 3–5 days.

Data on prophylactic negative pressure wound therapy after CD are limited. A systematic review of 7 randomized trials using negative pressure wound therapy in patients with chronic open wound compared with hydrocolloid gel plus gauze or gauze soaked in normal saline or Ringer’s solution did not shown improvement in wound healing [60]. Retrospective studies of prophylactic negative pressure wound therapy after CD in morbidly obese women, however, demonstrated fewer wound complications [61, 62]. A cost-benefit analysis showed that negative pressure wound therapy is only beneficial if the risk of surgical site infection is greater than 14% [63]. A large randomized controlled trials comparing prophylactic negative pressure wound therapy with standard dressing in women with obesity would be useful.

## Postoperative management

### Dressing removal between 24 and 48 h

Centers for Disease Control and Prevention recommend dressing removal between 24 and 48 h [64]. In a group of women who underwent scheduled CD, Peleg et al. in 2016 conducted a randomized controlled trial comparing postoperative dressing removal at 6 h (*n* = 160) with dressing removal at 24 h (*n* = 160) and showed no difference in wound complications [7]. However, the women with earlier dressing removal were more pleased or satisfied than those with later dressing removal (OR 2.35; 95% CI 1.46, 3.79). It is unknown if timing of dressing removal would make a difference in women having emergent CD or those with comorbidities such as obesity, diabetes, or hypertension.

### Daily use of chlorhexidine gluconate soap after removal of dressing

Information on daily use of chlorhexidine gluconate soap after dressing removal in women after CD is limited. In a randomized nonblinded crossover trial of 7727 patients admitted in intensive care and bone marrow transplantation units, daily bathing with chlorhexidine-impregnated washcloths had a lower risk of acquisition of multidrug-resistant organisms (5.10 cases per 1000 patient days vs. 6.60 cases per 1000 patient-days; *P* = .03) and hospital acquired bloodstream infections (4.78 cases per 1000 patient days vs. 6.60 cases per 1000 patient-days; *P* = .007) [65]. Similar studies involving post-CD patients warrants further study.

## Surgical bundle

Perioperative bundles of evidence-based practices to reduce SSIs have been introduced into non-obstetric surgical patients with promising results [66–68]. Similar results can be anticipated in the obstetric population. We recommend the development of SSI bundles for each hospital. Our own institution, MedStar Washington Hospital Center, began implementing a SSI bundle in December 2016 (Table 3). The study comparing the rate of SSIs prior to and after bundle implementation is underway.

**Table 3:** Surgical site infection bundle at MedStar Washington Hospital Center

Perioperative elements
Preoperative standard antibiotics
Preoperative intravenous azithromycin 500 mg
Chlorhexidine alcohol skin preparation
Use of clippers instead of razor
Vaginal cleansing by povidone-iodine
Intraoperative elements
Removal of placenta by traction of umbilical cord
Suture closure of subcutaneous tissue if wound thickness greater than 2 cm
Suture skin closure instead of staple closure
Postoperative elements
Dressing removal between 24 and 48 h
Daily use of chlorhexidine gluconate soap after removal of dressing

## Treatment

### Management of wound hematoma and seroma

Small hematomas may resorb without surgical interventions, although they increase the incidence of SSI. Management of wound hematoma includes evacuation of the clot under sterile conditions, ligation or cauterization of bleeding vessels, and reclosure of the wound [69].

Seromas delay wound healing and increase the risk of SSI. Seromas under skin can be evacuated by needle aspiration. To prevent reaccumulation, compression dressings should be applied. If seromas persist, wound exploration in the operating room may be required [69].

### Management of wound infection

Management of wound infection includes antibiotics, incision and drainage, wound dressing, and delayed closure.

#### Antibiotics

Superficial infection such as cellulitis can be treated with antibiotics alone and do not require incision and drainage. If purulent drainage or exudates accompany cellulitis, empiric therapy should include adequate coverage for methicillin-resistant *Staphylococcus aureus* (MRSA) [70]. Options for oral antibiotics include clindamycin, trimethoprim-sulfamethoxazole, and tetracycline (doxycycline or monocycline). If cellulitis is nonpurulent (no purulent drainage or exudate and no abscess), empiric therapy to cover beta-hemolytic streptococci and methicillin-sensitive *Staphylococcus aureus* (MSSA) is recommended [71]. Options for oral antibiotics for nonpurulent cellulitis include dicloxacillin, cefadroxil, cephalexin, and clindamycin.

#### Incision and drainage

If the wound has purulent drainage, exudate or separation, incision and drainage to remove abscess, exudate, and hematoma is needed. If necrotic tissue is identified, sharp debridement using forceps and scalpel or scissors is needed until healthy tissue can be identified [72]. Further wound exploration to confirm the integrity of fascia is also important. Fascial dehiscence is a surgical emergency and requires further wound exploration in the Operative Room.

#### Wound dressings

Packing may be required if the wound has a deep defect. Commonly, wet to dry dressing changes several times daily are performed, placing moistened gauze into the wound with covering by dry gauze [73]. When the gauze is removed during the dressing change, necrotic tissue is also removed. Once necrotic tissue is completely removed and healthy granulation tissue starts to grow, dressing changes can be done less frequently. Other available dressing materials include foam, beads, alignate, hydrocolloids, and gauze with chlorhexidine, povidone iodine and mercury chloride antiseptic solution. A systematic review showed no difference in speed of wound recovery using these various types of dressing materials [74].

### Delayed closure

Infected wounds should be left open to heal by secondary intention. In review of 8 prospective studies, reclosure of wound was associated with 81-100% successful healing [75]. Failures occurred in 21 of 324 reclosed wound, 16 of which were complicated by recurrent abscesses. Wound healing was faster in reclosure group compared with secondary intention (16–18 days vs. 61–72 days).

### Management of necrotizing fasciitis

Treatment of necrotizing fasciitis includes early and aggressive surgical exploration and debridement of necrotic tissue in addition to broad-spectrum antibiotic therapy [76]. The goal of surgical management is debridement of necrotic tissue until healthy, viable tissue is reached. In most cases, re-exploration of wound 24–36 h after the first debridement and daily thereafter is necessary until no necrotic tissue is found.

Empiric treatment of necrotizing fasciitis should include agents effective against aerobes, including methicillin-resistant *Staphylococcus aureus*, and anaerobes. Acceptable choices for antibiotics are vancomycin, linezolid, or daptomycin combined with one of the following options 1) piperacillin-tazobactam, 2) carbapenem, 3) ceftriaxone plus metronidazole, or 4) fluoroquinolon plus metronidazole [76]. In the setting of group A streptococcal or beta-hemolytic streptococcal infection, the antibiotic regimen should be narrowed down to the combination of penicillin (4 million units every 4 h) and clindamycin (600-900 mg every 8 h). Clindamycin suppresses streptococcal toxin and cytokine production. The efficacy of intravenous immunoglobulin (IVIG) to neutralize extracellular streptococcal toxins is unclear. In a randomized controlled trial in Europe, addition of IVIG to surgical treatment and antibiotics therapy did not improve survival in women with streptococcal toxic shock syndrome [77]. In the setting of Clostridium infection, penicillin plus clindamycin is recommended [78]. The efficacy of hyperbaric oxygen therapy for clostridium infection is unclear due to the lack of data from randomized controlled trials in humans.

### Management of endometritis

Endometritis is generally treated by clindamycin (900 mg intravenously every 8 h) plus gentamicin [79]. Gentamicin (5 mg/kg [ideal body weight]) compared with gentamicin (1.5 mg/kg [ideal body weight] every 8 h) is similarly efficacious, more cost effective and less task time for nurses [80–82]. Ampicillin may be added to the regimen for better coverage of enterococcus. If fever persists despite antibiotics administration, imaging of abdomen and pelvis should be considered to rule out infected hematoma and pelvic abscess.

## Conclusions

SSIs following CDs represent complex clinical situations and are caused by many factors such as patient characteristics and perioperative management. In addition, SSIs comprise a burden to our health care system. Creating bundles of evidence-based elements may decrease the rates of post-CD SSIs, as has been demonstrated in non-obstetric patients. We strongly recommend each hospital to consider the evidence-based information presented in creating its own surgical bundle to decrease the rates of SSIs after CDs.

## Abbreviations

ACOG: American College of Obstetricians and Gynecologists
BMI: Body Mass Index
CI: Confidence interval
OR: Odds ratio
RR: Relative Risk
